# Association of Shift Patterns with Overwork, Turnover, and Care Quality in U.S. Skilled Nursing Facilities

**DOI:** 10.1093/geroni/igaf122.2420

**Published:** 2025-12-31

**Authors:** Fangli Geng, Yuqian Lin, Pedro Gozalo, Elizabeth White

**Affiliations:** Brown University, Providence, Rhode Island, United States; Massachusetts General Hospital, Boston, Massachusetts, United States; Brown University, Providence, Rhode Island, United States; Brown University School of Public Health, Providence, Rhode Island, United States

## Abstract

Workforce challenges, including high turnover and staffing shortages, persist in skilled nursing facilities (SNFs). Understanding how shift structures influence workforce stability and care quality is critical for policy and operational decisions. This study identified the dominant shift for SNFs, defined as the most frequent daily work hours for the majority of nurses (Registered Nurses, Licensed Practical Nurses, and Certified Nurse Assistants), and classified SNFs into three shift categories: predominantly 8-hour shifts, 12-hour shifts, or a mix of both. Using facility-level data from 2021, integrating Payroll-Based Journal data, Nursing Home Care Compare quality measures, and Long-Term Care Focus facility characteristics, we fit linear regression models with state fixed effects to assess the association between dominant shift type and key workforce and care quality outcomes, adjusting for facility characteristics such as ownership, size, and case mix. Of the total 13,071 facilities studied, 10,821 (83%) had a dominant 8-hour shift, 1,918 (15%) had a dominant 12-hour shift, and 332 (2%) used mixed shifts. Facilities with 12-hour or mixed-shifts had significantly higher overwork prevalence (2.1 and 1.3 percentage points higher than 8-hour facilities, respectively) and turnover rates (7.6 percentage points higher for 12-hour shifts). Quality of care was also worse in 12-hour shift facilities across 16 of 22 measures, including higher rates of pressure ulcers, falls, and depressive symptoms. Policies should address the implications of different types of shifts, and future research should explore interventions to balance staffing efficiency with staff well-being and patient care.

